# Food Reformulation in New Zealand: A Success Story of Reducing the Sodium Content in Bread from 2003 to 2023

**DOI:** 10.3390/nu17223627

**Published:** 2025-11-20

**Authors:** David Monro, Nan Hu, Rachael Mira McLean

**Affiliations:** 1 The National Heart Foundation of New Zealand, Auckland 1546, New Zealand; davem@heartfoundation.org.nz; 2 Department of Public Health Dunedin, University of Otago, Dunedin 9054, New Zealand; nan.hu@postgrad.otago.ac.nz

**Keywords:** bread, salt, sodium, salt reduction, food reformulation, New Zealand

## Abstract

Background: The National Heart Foundation of New Zealand (HF) has successfully supported bread companies to reduce the sodium content in leading selling breads over the past 20 years. Bread is the main source of sodium in the New Zealand (NZ) diet and is considered a low-cost staple food for many New Zealanders. Methods: We highlight some of the critical success factors in developing a food reformulation programme in NZ, using the changes in the packaged loaf bread category as an example. The research considers sodium reduction through three different approaches: (1) matched products, (2) averages of products and (3) sodium contents of the top-selling breads in 2023. Results: The biggest sodium reductions were for white breads, where the mean sodium content dropped from 517 mg/100 g in 2003 to 389 mg/100 g in 2023, representing a 25% reduction. White breads are priced lower than other breads, highlighting the programme’s impact on health equity. The mean sodium content of all breads involved in the study in 2003 was 472 mg/100 g and in 2023 was 384 mg/100 g, representing a 19% reduction overall in the mean sodium content per 100 g. The mean sodium content for the top 20 breads by sales volume in 2023 was 382 mg/100 g, indicating that companies had reduced sodium in leading selling products and the changes had not compromised sales. Conclusions: A key factor for the success of this salt reduction work is the long-standing relationship between a trusted health organisation (HF) and major bread companies. Sodium targets are set in consultation with key food companies and consider any technical and commercial constraints. Government funding has ensured a long-standing programme of work where trust is built with companies, and targets can be revised and monitored. The opportunity from here is to explore where further reductions can be made in the bread category and where these results can continue to drive success in other food categories.

## 1. Introduction

### 1.1. Salt, Blood Pressure

High salt (sodium) intakes are associated with elevated blood pressure, a leading risk factor for early death globally. A high salt intake is associated with important cardiovascular disease outcomes such as acute coronary syndrome and stroke, as well as an elevated risk of stomach cancer [1]. It is estimated that NZ adults consume a mean of 8.4 g salt per day (3373 mg sodium) [2], which is well above the World Health Organization (WHO) guideline (<5 g salt or <2000 mg sodium per day) [3]. A reduction in population salt intakes by 30% by 2025 is one of nine global targets agreed to by 194 WHO Member States including NZ [4].

### 1.2. Food Reformulation and the Heart Foundation’s Work with Food Companies

In a Western diet, the majority (70–80%) of dietary salt consumed by the population comes from processed foods or restaurant foods [5]. The reformulation of processed foods to reduce salt content is a key strategy for salt reduction in countries where a substantial proportion of intake comes from packaged, processed and restaurant foods, because it reduces salt intake without requiring individual behaviour changes. It is a pro-equity approach as it targets the whole population, especially those who rely on low-cost processed foods, rather than just those who can afford healthier, more expensive alternatives. Of the 194 WHO Member States, 34% (*n* = 65) have implemented policies to reformulate manufactured food to contain less sodium. Bread and bread products are the most targeted food category for sodium reduction, followed by processed meat, poultry, game or fish; ready-made and convenience foods and composite dishes; and savoury snacks [6]. Many countries (e.g., the United Kingdom, Australia, Canada and the United States) have voluntary salt reduction strategies in place and have demonstrated that reductions of between 9% and 30% are possible in breads [7]. Furthermore, reduction in salt intake through food reformulation and the setting of target levels for the amount of salt in foods is identified as a ‘best buy’ in terms of cost-effective interventions for reducing unhealthy diets to prevent noncommunicable diseases [8].

The HF has had a long-standing philosophy of working with food companies as a critical part of salt reduction in the population [9,10]. HF efforts have been directed at food categories that are leading sources of salt in the NZ diet and priority is given to working with major companies who can reformulate the highest-selling products and bring the largest public health impact.

In order to maximise the equity of health outcomes, the HF prioritises lower-cost products as part of its engagement with food companies. Foods from supermarkets are an essential purchase for all New Zealanders, and twenty-two food companies dominate the NZ packaged food market [11]. Supermarkets report that many shoppers (40%) are switching to cheaper brands as part of coping with the increased food costs [12]. In 2024, the price of groceries was consumers’ biggest financial pressure [13] and had grown significantly as a concern since 2021 [14]. Therefore, improving the nutritional quality of lower-cost foods has become increasingly important over the past 5 years as the cost of food has significantly increased in New Zealand [15,16].

### 1.3. Bread Is a Staple Food in New Zealand

Bread is a relatively low-cost staple food in NZ, with the most recent national Adult Nutrition Survey showing it contributed on average to 11% of total energy intake for those aged 15 years and above [17]. Most bread is sold through supermarkets [18], with loaf bread, (particularly white loaf bread), being amongst the top selling items for NZ shoppers [19]. The ‘bread’ category has also been reported to account for eight of the top 20 selling products in supermarkets [20].

There are two major bread manufacturers in NZ—Goodman Fielder [21] and George Weston Foods [22], with almost all the bread consumed in NZ coming from these two companies in the form of their own brands or labelled under supermarket-own brands (or private labels) [23,24].

Bread is also the leading source of salt in the diets of New Zealanders, estimated to account for 35–43% of total salt intake, followed by other common sources such as sausages, meat pies, pizza, instant noodles, cheese, biscuits, potato crisps and tomato sauce [25]. Salt is one of the four key ingredients used to make bread (flour, water, yeast and salt) meaning that modifying it can be a difficult task. Salt in bread modifies flavour, increases crust colour and controls the rate of yeast fermentation and enzyme activity [26]. If yeast fermentation happens too quickly, then the dough can become gassy and sour and can have poor structure and volume. It also means that the dough becomes slack, sticky and weak and creates major processing issues leading to a poor-quality final product [27,28,29]. Salt is also added to bread as a preservative to delay mould and yeast growth on the surface of bread during its shelf-life [28,30]. This is particularly important if the bread is manufactured and then needs to maintain its shelf life in humid conditions. (e.g., in some of New Zealand’s largest cities).

### 1.4. Research and Target Development in the NZ Bread Category

Research in 2003 compared the sodium contents of lower-cost vs. higher-cost products available across nine packaged food categories including bread. Lower-cost breads had higher sodium contents relative to higher-cost breads. The majority of these lower-cost breads were white breads and had sodium contents of over 500 mg/100 g [31].

In 2007, the HF was funded by the NZ Government to develop and pilot a salt reduction target for the packaged loaf bread category. A sodium target of 450 mg/100 g was developed in consultation with major bread companies. The target was based on the sodium contents in bread from the 2003 research, existing sodium contents in NZ bread in 2007 and feedback from companies on any technical constraints. The target was implemented by the two major bread companies and the two private label companies over a one-year period. The reformulations made resulted in an 18% average sodium reduction in white breads, and 150 tonnes of salt removed from these breads in one year [32].

The sodium reduction targets for bread were revised from 400 mg/100 g in 2014, to 380 mg/100 g in 2018 and 370 mg/100 g in 2023 based on sodium contents in the market and consultation with key food companies. The bread targets were expanded in 2018 from leavened loaf bread to include a flat bread, tortillas and pita breads as a subcategory given the growing popularity of these products [33].

This paper will explore whether the sodium content of individual formulations changed over the time period, and also the impact of reformulation on the highest-selling supermarket breads available on the market in 2023. It will also discuss success factors for working with the bread industry on a salt reduction model, and the considerations in developing targets for food reformulation.

### 1.5. Aim of Study

The aim of this study was to describe and evaluate the outcomes of the HF bread reformulation programme and its impact on the sodium content in NZ bread from 2003 to 2023.

## 2. Methods

Sodium contents were obtained for white breads and wholemeal/wheatmeal/grain leavened packaged loaf breads available in NZ in 2003, 2013 and 2023.

### 2.1. Data Collection

Data Collection 2003—data on the sodium content of food products was collected by the research team from the four major supermarkets in Dunedin, NZ [31]. Sodium values were collected directly from the Nutrition Information Panels on product packages. Data were collected for nine food categories with bread being one of these categories. The lead researcher cross checked the nutrition data for accuracy.

Data collection 2013—companies provided sodium data as part of their annual reporting to the HF. All major bread companies and retailers were involved in the programme, meaning that the bread market was well covered. The information obtained was verified by checking the Nutrition Information Panels on product packages at four major supermarkets in Auckland, NZ and values present on food company websites (where they were available).

Data collection 2023—sodium contents were obtained from GS1 NZ [34]. GS1 collects product information such as barcode information, alongside nutritional information, allergen information and use of the Health Star Rating for all supermarket products. The information is stored in a secure database. The HF purchases the access to the database for specific food categories. This information is used for the purposes of food reformulation target setting and ongoing monitoring.

Collecting sodium information from product packaging and using databases for accessing this type of information is a commonly used approach for this type of research [35,36]. Furthermore, label information is often a more uniform measure than nutritional analysis due to the variances that can occur with analysis.

### 2.2. Data Cleaning and Matching

Data sets were collated and categorised into white breads and wholemeal/wheatmeal/grain breads. The focus of the paper was to examine changes in high-volume low-cost wheat-based packaged loaf breads, which were priority products when developing salt reduction targets. The following bread types were therefore excluded from the data set: gluten free breads and buns, muffins, hot cross buns, burger buns, paleo breads, keto breads, artisan breads, bagels, fruit breads, flavoured breads, and hamburger buns and rolls.

Included in this analysis were all high-volume breads that existed on the market over the time period (matched breads). These bread brands were matched by brand and company name across 2003, 2013 and 2023 time points. Where there had been a name change in the product, an enquiry was made with the food companies to check if it represented the same product.

If a product had several different variants (for example, thin slice, sandwich slice, toast slice and super-thick slice bread) only one formulation was selected for the creation of the averages for each time period and making the comparisons in 2003, 2013 and 2023. This was to prevent the averages and the data set from being skewed by certain bread brands having multiple entries. The lead researchers checked that the sodium content for these different variants was the same to ensure that they were the same formulation.

### 2.3. Sales Data

Sales data for 2023 was obtained for the bread category from Circana (NZ) Limited. Circana is a leading market research company that offers sales insights to brands and retailers across various industries (including the food and beverage industry). The sales data were provided in adherence to retailer data sharing rules and enabled the assessment of whether sodium reductions were being made in the highest volume, leading selling breads in the category. The mean sodium content and the sales-weighted means for the top 5, 10 and 20 selling breads was calculated along with the mean of the top 10 selling white breads and top 10 selling wholemeal/wheatmeal/grain breads. Where there were multiple formulations, they were not combined. Sales data were only assessed for 2023 as they were not available for 2003 and 2013. Sales-weighted means were created for 2023 by multiplying each bread’s sodium by the units sold then adding these numbers together to obtain a weighted total. The weighted total was then divided by the total units sold.

### 2.4. Analysis

Data were collected into an Excel spreadsheet for descriptive analysis.

The key assessments included the following:

- Sodium changes in matched bread brands between 2003, 2013 and 2023 along with a percentage reduction over each time period and overall (2003 to 2023). Mean sodium content (mg/100 g), 2003, 2013 and 2023, mean change and mean percentage reduction.
- Mean sodium content for white breads and wholemeal/wheatmeal/grain breads in 2003, 2013 and 2023. Sodium changes (mg/100 g) along with percentage reduction between each year and overall (2003 to 2023).
- Mean sodium content and sales-weighted means of the top 20 selling breads, the top 10 selling white breads and the top 10 wholemeal/wheatmeal/grain breads in 2023.

## 3. Results

**Figure 1:**
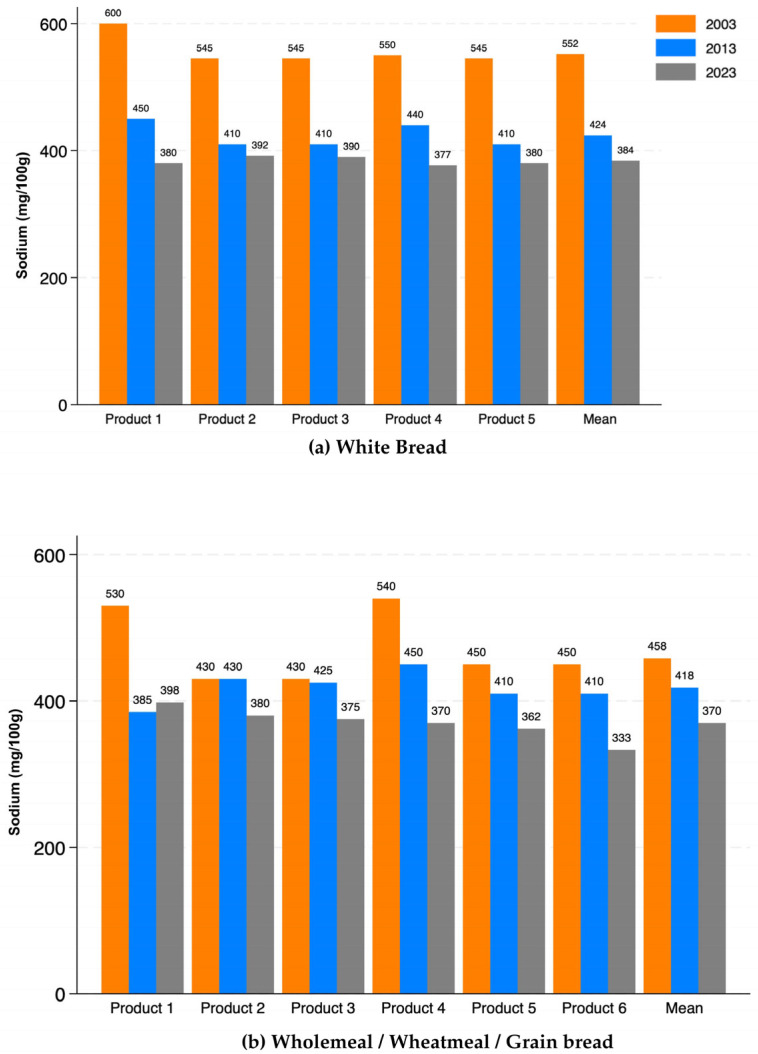
Sodium content (mg/100 g) of bread products at three time points (2003, 2013 and 2023). (**a**) White bread: mean sodium levels across five matched products and the overall mean. (**b**) Wholemeal/wheatmeal/grain bread: mean sodium levels across six matched products and the overall mean.

The mean sodium content of matched white breads (*n* = 5) decreased over the period from 552 mg/100 g in 2003, to 424 mg/100 g in 2013 and 384 mg/100 g in 2023. This represented a 24% reduction between 2003 and 2013 and a 9% reduction between 2013 and 2023 with a 31% reduction overall between 2003 and 2023. There was a smaller reduction in the mean sodium content of wholemeal/wheatmeal/grain breads that were available in the 2003, 2013 and 2023 datasets (*n* = 6) (see Table 1 and Figure 1). The mean sodium content decreased from 458 mg/100 g to 418 mg/100 g to 370 mg/100 g, respectively, a reduction of 10% between 2003 and 2013 and an 11% reduction between 2013 and 2023 with a 21% reduction overall between 2003 and 2023.

The mean sodium content of all breads in 2003, 2013 and 2023 was 472 mg/100 g (*n* = 44), 422 mg/100 g (*n* = 109), and 384 mg/100 g (*n* = 160), respectively (see Table 2). These values represent an 11% reduction between 2003 and 2013 and a 9% reduction between 2013 and 2023, with a 19% reduction overall between 2003 and 2023. White breads showed the biggest reductions with the mean sodium content reduced from 517 mg/100 g (*n* = 16) in 2003 to 389 mg/100 g in 2013 (*n*= 35), representing a 25% reduction over the 20-year period. Wholemeal/wheatmeal/grain breads had a reduction from 447 mg/100 g (*n* = 28) in 2003 to 383 mg/100 g (*n* = 125) in 2023, representing a 14% reduction over the 20-year period.

The mean sodium content of the top 5, 10 and 20 top-selling breads are outlined in Table 3. The mean sodium content of the top 5 and 10 top-selling breads in 2023 was 391 mg/100 g and 390 mg/100 g, respectively. When this was expanded out to the top 20 breads, the sodium content was lower, at 382 mg/100 g. The top 10 selling white breads had an average sodium content of 390 mg/100 g, which was higher than the top 10 selling wholemeal/wheatmeal/grain breads which had an average sodium content of 380 mg/100 g.

## 4. Discussion

The results of this study demonstrate substantial sodium reductions in commonly purchased bread products in NZ over a 20-year period. The mean sodium content of breads reduced from 472 mg/100 g in 2003, to 384 mg/100 g in 2023. This represented a 19% reduction overall in the average sodium content per 100 g from 2003 to 2023. The biggest reductions were for white breads where the average sodium content dropped from 517 mg/100 g in 2003 to 389 mg/100 g in 2023, representing a 25% reduction over that period. White breads had a larger reduction compared with wholemeal/wheatmeal/grain breads (14% reduction), because white breads started from a higher sodium content in 2003. For products that could be matched across the three time points, there was a 31% reduction in the average sodium content of matched white breads (*n* = 5) and a 21% reduction in the sodium content of matched wholemeal/wheatmeal/grain breads (*n* = 6). This represents a substantial reduction in sodium content across the range of packaged loaf breads available in NZ supermarkets over the period. Sales data are not available for the 2003 and 2013 time points; however, the reductions made to matched products available in the market for 20 years indicate they are likely to be some of the leading selling and most consumed breads across the NZ population. Sales data for 2023 provide an indication of consumer exposure at the most recent point. The average sodium content for the top 5, top 10 and top 20 breads by sales volume in 2023 was 391 mg/100 g, 390 mg/100 g and 382 mg/100 g, respectively, indicating that companies had reduced sodium in leading selling products. This finding was echoed when the data were separated into the top 10 selling white breads (390 mg/100 g) and wheatmeal/wholemeal/grain breads (380 mg/100 g). Sales-weighted means were developed; however, they showed little difference compared with the mean.

### 4.1. Salt Reduction

Bread companies have reduced the percentage of salt added into their bread recipes over the 20-year period without using salt replacers, added sugar or other preservatives. Reducing the percentage of ingoing salt is considered the most simple and cost-effective method that is used by the bread companies to reduce the sodium content of breads [37].

These results demonstrate that the partnership programme between the HF and food industry stakeholders has led to substantial reformulation efforts by bread companies, particularly the two major companies and supermarket-own brands. Bread is a staple food for New Zealanders and is the leading source of salt in the NZ diet [25]; the breads included in the programme made up a large proportion of the bread sold through supermarkets and consumed by New Zealanders.

The reductions in the sodium content of white bread are important from an equity perspective. The 2003 research had shown that white breads tended to be higher in sodium compared with wheatmeal/wholemeal/grain breads (and are also on average priced significantly lower than many wholemeal/wheatmeal/grain breads [31]. In the wake of high food costs in NZ and cost of living challenges, the highest volume and lowest cost foods within a food category need to be at the heart of reformulation efforts by food companies and the focus of public health stakeholders.

The setting of sodium reformulation targets is widely regarded as a best practice for public health strategies to reduce dietary sodium [7,38]. The successful United Kingdom Food Standards Agency’s salt reduction programme implemented a series of reduction targets over a nearly 20-year period in recognition that stepwise reductions were less likely to be noticed by consumers than large reductions over a short time period [39,40]. Between 2001 and 2011, the programme achieved a 20% sodium reduction in the sodium content of bread, further supporting the effectiveness of a target-based approach to salt reduction [41].

This paper highlights that in NZ, substantial reductions in sodium content in bread were made in a stepwise fashion, without compromising acceptance. This is consistent with research that demonstrates that when sodium contents are reduced gradually in steps of 10–20%, that consumers do not detect the change and there is good consumer acceptance [42,43,44]. The average sodium content of the top-selling breads also indicates that salt reduction has been made without compromising sales.

### 4.2. Success Factors for Salt Reduction in Breads

A key factor to the success of this salt reduction work is the long-standing relationship between a trusted non-government organisation (HF) and major bread companies. The current programme has been running since 2007, enabling long-term collaborative relationships to be developed [32]. Critical to this approach is seeking an understanding of food company perspectives and constraints and developing workable and realistic solutions in partnership with them, which can provide a win–win for health and the food industry.

The most common approach to salt reduction globally is a voluntary target, with some level of government involvement [7,45]. Government funding of this reformulation programme in NZ has ensured a continual programme of work that provides food companies confidence in the work plan, allows relationships to be built, trust to be gained and for the ongoing revision and monitoring of the targets over time.

Food reformulation can often take several years, as food companies will not alter the formulation of products every year. Instead, changes are made over a longer period where companies are able to apply stepwise reductions, and salt reduction can be tied in with other changes to reduce business costs [5]. These other changes might include a change in ingredients to reduce costs or updates to packaging. The targets that have been set in NZ for the bread category (and other categories) have a 4 to 5-year time frame [46]. This follows international best practice and is consistent with other food reformulation programmes globally [41].

### 4.3. Target Development

The bread targets for NZ are set by the HF in consultation with leading food companies, and often the nutrition, technical, and quality assurance areas of the business are the key contact points for the HF. Determining sodium levels in the NZ market at a specific time point, alongside any technical and commercial constraints highlighted by food companies, has been critical to target development. The bread targets in 2007 were initially designed to have a greater focus on salt reduction in white bread. Over time, the targets were lowered to encourage further salt reduction across all breads and also expanded to include other types of breads (wraps and pita breads) which were growing in popularity. A key aspect of companies reducing sodium content is to have the food reformulation targets agreed to by all the major bread companies, and subsequently have the companies reduce salt content at the same time so that a company is not placed at a commercial disadvantage.

Bread is the most targeted food category globally for food reformulation with most high-income countries having salt reduction targets for the product [7,47]. Most western countries who have bread targets have sodium contents between 350 mg/100 g and 450 mg/100 g [47]. The WHO developed a set of sodium benchmarks in 70 food subcategories to assist member countries to develop salt reduction targets. Sodium targets were looked at from 41 different countries in developing these benchmarks. The second edition of these benchmarks was released in April 2024 and the NZ bread target of 370 mg was adopted as the global benchmark [38].

Considering global benchmarks and targets set in other markets (Table S1) is an important consideration in setting national targets [47]. However, the major factor that has guided successful target development for the NZ market is the sodium content in a food category at that time point, particularly the content in high-volume lower-cost foods that need to be a focus of salt reduction. The initial NZ bread target of 450 mg/100 g in 2007 was set 50 mg above the Australian target [48]. This was because NZ breads were sitting at a much higher baseline level and there was a specific focus to encourage the reformulation of many white breads which were above 500 mg/100 g.

### 4.4. Further Sodium Reduction

The current reformulation target for the NZ loaf bread category set in 2023 is 370 mg/100 g [46]. The average for the top 10 selling wholemeal/wheatmeal/grain breads in 2023 is 380 mg/100 g, close to this 370 mg target, and the top 10 selling white breads are higher at 390 mg/100 g, still some way off the 370 mg/100 g target. This highlights the potential for further sodium reduction in the bread category towards the current NZ target. Expansion of sodium reduction across the bread category (including, for example, wraps and tortillas) would also be beneficial.

To reduce excessive intake of sodium from bread, a variety of methods has been investigated. Depending on the sodium content of the full-salt product typically 1–2% salt relative to flour weight—a reduction of 15–25% salt appears feasible for most types of bread, with some as significant as a 33% reduction [37]. Bread companies have highlighted the challenges of going below the current target of 370 mg—particularly around dealing with humid conditions in manufacturing and shelf life. If salt levels are too low, this creates a sticky dough, leading to difficulties in manufacturing and wastage. A lower percentage of salt can mean a reduction in shelf life and the possibility of increasing other preservatives, which goes against consumer preferences for a clean label and a more natural product. Potassium chloride is another approach that has been used as a part replacement for sodium chloride in bread. Some research has shown that up to a 50% replacement [49] is possible with consumer acceptability. However, other research has shown that up to 30% can be made; although, metallic flavours are detected by consumers when levels reach 40% [50,51]. Currently, there is not a wide use of potassium chloride through any high-selling breads in NZ according to common supermarket product listings [52,53,54]. If potassium chloride was to become more widely used, then safety and any negative impact particularly for children, pregnant women and those with impaired renal function would need to be assessed [55].

### 4.5. Other Approaches for Reducing Population Sodium Intake

This paper discusses salt reduction made in the bread category in NZ. The effect of the salt reduction in bread on blood pressure is difficult to quantity without up-to-date national nutrition survey data. Although bread is the largest contributor to salt intakes, there would need to be salt reduction across many food categories [47] and other significant changes to reduce New Zealand’s estimated salt intake of 8.4 g/day [1] to the WHO target of 5 g/day [2]. It has been estimated that a 36% reduction in the sodium content of packaged foods in conjunction with a 40% reduction in discretionary salt use and the sodium content of foods consumed away from the home would reduce total population salt intake in NZ by 35% (from 8.4 g to 5.5 g/day) [3]. The target for bread was 350 mg/100 g. The health benefits of such a change are large. Modelling in the United Kingdom has shown that reducing daily salt intake from 8.4 g to 6 g/day by 2025 to 5 g/by 2030 would result in 1.4 million fewer people living with hypertension in 2035 compared to the baseline scenario of intake staying the same; therefore, 134,789 cumulative incidences of coronary heart disease and 48,540 cumulative incidences of stroke could be avoided by 2035 [56].

Reformulation remains only one component of reducing the population’s salt intake. The WHO SHAKE technical package provides a national blueprint for sodium reduction and highlights the importance of other strategies sitting alongside a food reformulation approach. These strategies include consumer awareness, education, dietary change, labelling, policy/guidelines for certain sectors and effective monitoring [5]. Further salt reduction across the food supply could be supported with stronger public health action across all areas of the SHAKE package within NZ.

### 4.6. Future Research

Future research should explore the potential for potassium chloride replacement in bread, the potential of other salt replacers and options for overcoming processing challenges when a lower percentage salt is added to the recipe. Since 2010, the reformulation approach with bread has been expanded to more than 50 targets in NZ, covering more than 40 food categories. In 2016, the first sugar reduction targets were developed [57]. Opportunities exist to explore progress with salt reduction and sugar reduction in other food categories, particularly in relation to leading selling products.

### 4.7. Strengths and Limitations

This study has several strengths. It has comprehensive data collection over a 20-year period covering major brands and nutrient levels. This is particularly the case with companies providing the data directly in 2013 and the use of highly credible partners in GS1 NZ and Circana NZ in 2023 for both nutrient and sales data. It considers sodium reduction through three different approaches: (1) matched products, (2) averages of products, and (3) sodium contents of the top-selling breads in 2023. The analysis has prioritised the impact on health equity by examining the highest-selling and lower-cost breads available through supermarkets—particularly wheat-based packaged loaf bread. The data sets in 2003, 2013 and 2023 have been cleaned to align with products that have been the focus of sodium reduction work since 2007.

There are several limitations. With the most recent data collected to 2023, it is likely that further changes in the sodium content of breads have occurred since 2023. For example, in the top 5 selling breads, there is one product indicated at 430 mg/100 g in the upper range. Nutrient data via online shopping in 2025 indicates this product has its sodium content reduced to 380 mg/100 g. Furthermore, this study only looked at packaged wheat-based breads and the highest-selling breads through supermarkets. However, it does not consider the full bread supply which also includes artisan breads, instore bakery, wraps, tortillas and bagels, etc. However, these are not the highest-selling and lower-cost breads through supermarkets. Opportunities exist to undertake further research looking at sodium reduction within NZ food categories over the long-term.

## 5. Conclusions

The HF food reformulation programme has supported a 19% reduction in the sodium content of wheat-based supermarket loaf breads over the last 20 years. In particular, the largest reduction of 25% has been made in white, lower-cost breads, highlighting the programme’s impact on health equity. A key success of the voluntary programme is the long-standing funding by the NZ Government and the relationships that are built between a trusted non-government organisation (HF) and major bread companies. Targets for the bread category have been revised three times (450 mg/100 g–370 mg/100 g) between 2007 and 2023 with the input from major bread companies. Food company input aims to create a buy-in and develop a workable solution that has the biggest potential impact on the highest-volume products. The use of up-to-date NZ-specific nutrition data, sales data and technical and commercial factors are all critical components of target setting. The programme’s success is demonstrated by major bread companies reducing sodium content in the market-leading/highest-selling products and NZ’s target being incorporated as part of the WHO global sodium-benchmarks 2024. The opportunity from here is to explore where further reductions can be made in the bread category and where these findings can continue to drive success in other food categories.

## Figures and Tables

**Table 1:** Sodium (mg/100 g)-matched products level by year and percentage reduction between timepoints and overall.

Bread Type	Product	2003–2013	2013–2023	2003–2023
		% Reduction	% Reduction	% Reduction
White	Product 1	25%	16%	37%
	Product 2	25%	4%	28%
	Product 3	25%	5%	28%
	Product 4	20%	14%	31%
	Product 5	25%	7%	30%
	Mean	24%	9%	31%
Wholemeal/ Wheatmeal/ Grain bread	Product 1	27%	−3%	25%
	Product 2	0%	12%	12%
	Product 3	1%	12%	13%
	Product 4	17%	18%	31%
	Product 5	9%	12%	20%
	Product 6	9%	19%	26%
	Mean	10%	11%	21%
Mean Overall				26%

**Table 2:** Mean (range) sodium (mg/100 g) 2003–2013–2023 and percentage reduction between timepoints and overall.

		Overall	White Bread	Wholemeal /Wheatmeal /Grain Bread
2003	Sodium Mean (mg/100 g)	472, *n* = 44	517, *n* = 16	447, *n* = 28
	Sodium Range (mg/100 g)	(350–530)	(450–600)	(350–530)
	Median (mg/100 g)	450	530	450
2013	Sodium Mean (mg/100 g)	422, *n* = 109	425, *n* = 27	421, *n* = 82
	Sodium Range (mg/100 g)	(366–530)	(380–540)	(366–530)
	Median (mg/100 g)	416	410	420
2023	Sodium Mean (mg/100 g)	384, *n* = 160	389, *n* = 35	383, *n* = 125
	Sodium Range (mg/100 g)	(315–525)	(327–453)	(315–525)
	Median (mg/100 g)	380	380	380
2003–2013	% reduction	11%	18%	6%
2013–2023	% reduction	9%	8%	9%
2003–2023	% reduction	19%	25%	14%

**Table 3:** Mean (range) sodium (mg/100 g) of highest-selling breads in 2023.

Bread Type	Top Selling	Sodium Range (mg/100 g)	Sodium Mean (mg/100 g)	Sales Weighted Sodium Mean (mg/100 g)
Overall	Top 5	(375–430)	391	391
	Top 10	(370–430)	390	390
	Top 20	(309–450)	382	386
White	Top 10	(377–430)	390	392
Wholemeal/Wheatmeal/Grain	Top 10	(375–400)	380	381

## Data Availability

The original contributions presented in this study are included in the article/Supplementary Materials. Further inquiries can be directed to the corresponding author.

## Supplementary Materials

The following supporting information can be downloaded at: https://www.mdpi.com/article/10.3390/nu17223627/s1, Table S1: Examples of voluntary sodium targets for leavened breads. References [58,59,60,61,62,63] were cited in Supplementary Materials.

## Abbreviations

The following abbreviations are used in this manuscript:

HF	The National Heart Foundation of New Zealand
NZ	New Zealand
WHO	The World Health Organization
