# Supplementary material for: Food Reformulation in New Zealand: A Success Story of Reducing the Sodium Content in Bread from 2003 to 2023
Source: Nutrients. 2025 Nov 20;17(22):3627. doi: 10.3390/nu17223627 (PMC12655271; doi:10.3390/nu17223627)
Supplement: Supplementary file 1 [file nutrients-17-03627-s001.zip › nutrients-3968478-supplementary.pdf]

Table S1: Examples of voluntary sodium targets for leavened breads

	**Target (mg/100g)**
New Zealand	370
Australia	380
United Kingdom	340 * (405 #)
United States	320* (470 #)
Canada<br>- white<br>- wholewheat and grain	 360 * (520 #)<br>330 * (400)
Hong Kong<br>- white<br>- wholewheat and grain	 380 (490 #)<br>380 (470 #)

*Sales weighted mean; # Maximum.

Source NZ https://assets.heartfoundation.org.nz/documents/food-industry/food-reformulation/heart-foundation-food-categories-and-reformulation-targets-summary-aug-2025.pdf?mtime=1755639151&_gl=1*1nfe8m*_gcl_au*MTI3MjA5NDc5NC4xNzU2MTU0NDgx
Source Aust https://www.health.gov.au/sites/default/files/2024-02/partnership-reformulation-program-summary-of-food-categories-and-reformulation-targets.pdf
Source UK https://assets.publishing.service.gov.uk/media/5f5618c8d3bf7f4d75de6ff1/2024_salt_reduction_targets_070920-FINAL-1.pdf
Source USA https://www.fda.gov/media/180794/download?attachment
Source Canada https://www.canada.ca/en/health-canada/services/publications/food-nutrition/sodium-reduced-targets-2020-2025.html#a6
Source Hong kong https://www.cfs.gov.hk/english/programme/programme_rdss/Sodium_Voluntary_Sodium_Reduction_Target.html
